# Biodegradation of Volatile Organic Compounds and Their Effects on Biodegradability under Co-Existing Conditions

**DOI:** 10.1264/jsme2.ME16188

**Published:** 2017-09-27

**Authors:** Miho Yoshikawa, Ming Zhang, Koki Toyota

**Affiliations:** 1 Geological Survey of Japan, National Institute of Advanced Industrial Science and Technology (AIST) 1–1–1, Higashi, Tsukuba, Ibaraki 305–8567 Japan; 2 Graduate School of Bio-Applications and Systems Engineering, Tokyo University of Agriculture and Technology 2–24–16, Koganei, Tokyo 184–8588 Japan

**Keywords:** biodegradation, chlorinated ethene, BTEX, chlorinated methane, multiple VOCs

## Abstract

Volatile organic compounds (VOCs) are major pollutants that are found in contaminated sites, particularly in developed countries such as Japan. Various microorganisms that degrade individual VOCs have been reported, and genomic information related to their phylogenetic classification and VOC-degrading enzymes is available. However, the biodegradation of multiple VOCs remains a challenging issue. Practical sites, such as chemical factories, research facilities, and illegal dumping sites, are often contaminated with multiple VOCs. In order to investigate the potential of biodegrading multiple VOCs, we initially reviewed the biodegradation of individual VOCs. VOCs include chlorinated ethenes (tetrachloroethene, trichloroethene, dichloroethene, and vinyl chloride), BTEX (benzene, toluene, ethylbenzene, and xylene), and chlorinated methanes (carbon tetrachloride, chloroform, and dichloromethane). We also summarized essential information on the biodegradation of each kind of VOC under aerobic and anaerobic conditions, together with the microorganisms that are involved in VOC-degrading pathways. Interactions among multiple VOCs were then discussed based on concrete examples. Under conditions in which multiple VOCs co-exist, the biodegradation of a VOC may be constrained, enhanced, and/or unaffected by other compounds. Co-metabolism may enhance the degradation of other VOCs. In contrast, constraints are imposed by the toxicity of co-existing VOCs and their by-products, catabolite repression, or competition between VOC-degrading enzymes. This review provides fundamental, but systematic information for designing strategies for the bioremediation of multiple VOCs, as well as information on the role of key microorganisms that degrade VOCs.

Volatile organic compounds (VOCs) are major pollutants that are found in soil and groundwater in developed countries. Contamination by tetrachloroethene (PCE), trichloroethene (TCE), benzene, and *cis*-dichloroethene (*cis*-DCE) accounts for approximately 11%, 10%, 9%, and 8%, respectively, in areas in which contamination exceeds environmental standards in Japan (121). In the United States, contamination by TCE, vinyl chloride (VC), benzene, and PCE accounts for 22%, 9%, 8%, and 7%, respectively, in the operable units of superfund sites (182). The International Agency for Research on Cancer reported the carcinogenic properties of VOCs, and, among them, TCE, VC, and benzene are associated with high cancer risks to humans (http://monographs.iarc.fr/). Thus, soil and groundwater that are contaminated with VOCs require remediation.

Regarding remediation technologies, bioremediation, which uses the degradation abilities of microorganisms, has received much attention because it is inexpensive, environmentally friendly, and applicable *in situ* (77, 210). According to a report by the United States Environmental Protection Agency (181), bioremediation accounted for 24% of the remediation technologies for contaminated groundwater. Various environmental microorganisms that are capable of degrading individual VOCs have been reported, and genomic information related to their phylogenetic classification and VOC-degrading enzymes are also available.

However, actual soil and groundwater, *e.g.*, those of chemical factories (139, 149), research facilities (176, 177, 179), military bases (178, 183), landfills (35, 184, 185), and illegal dumping sites (180), are frequently contaminated with multiple pollutants rather than a single type of VOC. Difficulties are associated with the biodegradation of multiple VOCs, which has remained a challenging issue in practice for decades (193). Alexander (4) reported that the effects of one VOC on other co-existing VOCs are largely unknown, and these effects have rarely been examined. Yoshikawa *et al.* (208) recently described a successful case study on the complete biodegradation of multiple VOCs including chlorinated ethenes, benzene, toluene, and dichloromethane through integrated anaerobic-aerobic biodegradation.

In order to systematically review the biodegradation of VOCs, and further investigate the potential of bioremediating multiple VOCs, we initially reviewed studies on the biodegradation of individual VOCs (Table 1), with an emphasis on information about useful microorganisms and mechanisms for the degradation of different VOCs. We investigated the biodegradation of chlorinated ethenes, BTEX (benzene, toluene, ethylbenzene, and xylene), and chlorinated methanes under aerobic and anaerobic conditions in detail. The effects of microorganisms on the biodegradation of a certain VOC with the co-existence of other VOCs were then evaluated in order to discuss the potential of bioremediation for multiple VOCs.

## Biodegradation of chlorinated ethenes

### Aerobic biodegradation of chlorinated ethenes

**Fig. 1:**
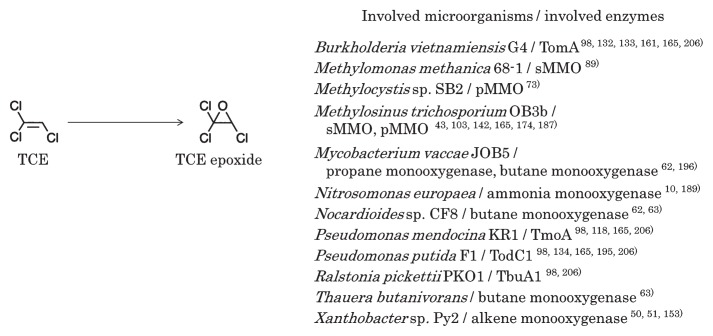
Possible initial step in the aerobic biodegradation of trichloroethene. TCE denotes trichloroethene. Abbreviations of involved enzymes indicate the following: TomA, toluene 2-monooxygenase; sMMO, soluble methane monooxygenase; pMMO, particulate methane monooxygenase; TmoA, toluene-4-monooxygenase; TodC1, toluene 2,3-dioxygenase; TbuA1, toluene 3-monooxygenase.

The aerobic biodegradation of chlorinated ethenes with natural gas containing methane, which acts as a co-substrate, was first discovered in the 1980s (201). Besides methane, aromatic compounds (133, 152), alkanes (63, 191, 196), alkenes (50, 64, 192), and ammonia (10) have been confirmed as co-substrates for the degradation of chlorinated ethenes. In addition, phytochemicals from poplar (*Populus*) leaves also function as co-substrates, resulting in the degradation of TCE (78). Oxygenases that degrade co-substrates lead to the degradation of chlorinated ethenes to epoxide compounds (Fig. 1). The growth-linked oxidation of chlorinated ethenes has only been reported for *cis*-DCE and VC. Limited information is currently available on the aerobic degradation of PCE (155), and, thus, further studies are required.

Aerobic microorganisms that degrade chlorinated ethenes with oxygenases have been isolated. Methanotrophs such as *Methylomonas methanica* 68-1 (89), *Methylocystis* sp. SB2 (73), and *Methylosinus trichosporium* OB3b (142) use methane monooxygenases to degrade chlorinated ethenes. Aromatic compound degraders, such as *Burkholderia vietnamiensis* G4 (132) and *Pseudomonas putida* F1 (134), use toluene monooxygenases and dioxygenases to degrade TCE. *Nocardioides* sp. CF8 and *Thauera butanivorans* use butane monooxygenases to degrade TCE, *cis*-DCE, and VC (63). *Mycobacterium ethylenense* NBB4, which was isolated on ethene, degrades VC (113). In contrast to the microorganisms described above, *Mycobacterium aurum* L1 oxidizes VC with growth, and uses an alkene monooxygenase to degrade *cis*-DCE, *trans*-DCE, and 1,1-DCE without growth (64, 65). Two microbes, *Polaromonas* sp. JS666 (38) and *Rhodococcus jostii* RHA1 (8), are known to oxidize *cis*-DCE with growth.

### Anaerobic biodegradation of chlorinated ethenes

**Fig. 2:**
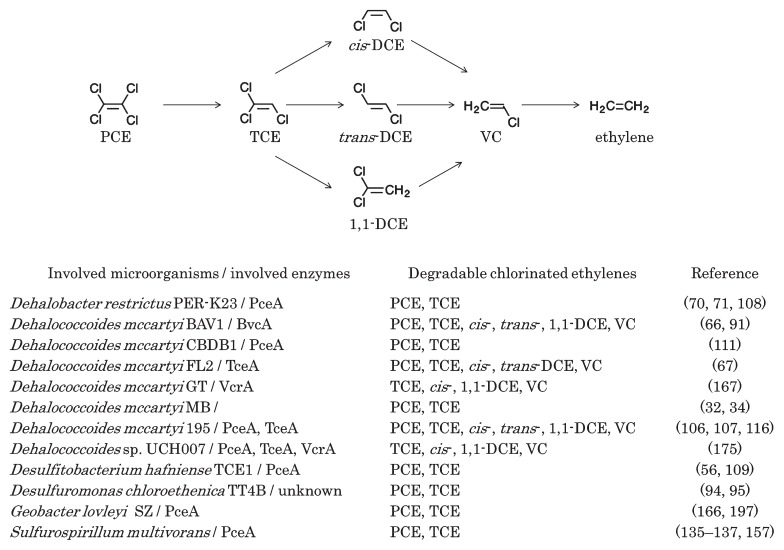
Possible pathways of anaerobic biodegradation for chlorinated ethenes. Abbreviations of involved enzymes indicate the following: PceA, dehalogenase dechlorinating PCE and TCE to *cis*-DCE; BvcA, dehalogenase dechlorinating VC; TceA, dehalogenase dechlorinating TCE to VC; VcrA, dehalogenase dechlorinating all DCE isomers to ethene. Abbreviations of VOCs indicate the following: PCE, tetrachloroethene; TCE, trichloroethene; DCE, dichloroethene; VC, vinyl chloride

The anaerobic biodegradation of chlorinated ethenes is caused by dechlorination, in which hydrogen sequentially displaces chlorine (186) (Fig. 2). PCE is mainly degraded to TCE, DCEs, VC, and harmless ethene, and among DCEs, *cis*-DCE predominates over *trans*-DCE and 1,1-DCE (164, 186). Dechlorination produces energy for degrading microbes; however, they cannot use chlorinated ethenes as a carbon source (86). Besides the main sequential dechlorination pathway described above, the anaerobic oxidization of *cis*-DCE, VC, and ethene have also been observed under sulfate-reducing and methanogenic conditions (22, 49, 115).

Anaerobic microbes that degrade chlorinated ethenes are diverse (Fig. 2). However, only the genus *Dehalococcoides* is known to degrade DCEs and VC. The isolation of anaerobic degraders of DCEs and VC has been a significant issue for a long time, and *Dehalococcoides mccartyi* 195 was first isolated in 1997 (102, 116). Strain 195 degrades PCE, TCE, *cis*-DCE, and 1,1-DCE as growth-linked substrates, and degrades *trans*-DCE and VC as non-growth substrates. Unlike other *Dehalococcoides* species, *D. mccartyi* strains MB and CBDB1 dechlorinate TCE and generate *trans*-DCE, rather than *cis*-DCE (32, 111). *Dehalococcoides* has key reductive dehalogenases, such as TceA, which dechlorinate TCE and all DCE isomers to VC, as well as VC to ethene at low dechlorinating rates (107), VcrA, which dechlorinates all DCE isomers to ethene, as well as TCE to *cis*-DCE at low dechlorinating rates. (129), and BvcA, which dechlorinates all DCE isomers to VC, and dechlorinates TCE without growth (91, 171). A gene expression analysis suggested that the reductive dehalogenase gene *mbrA* is involved in the production of *trans*-DCE in the dechlorinating pathway (34). *Desulfitobacterium* strains as well as *Dehalococcoides*, have the dehalogenase, PceA, which dechlorinates PCE and TCE to *cis*-DCE (60, 168). Strains of *Dehalococcoides*, such as BTF08 highly enriched from groundwater and UCH007 isolated from groundwater in Japan, contain the genes of three well-known reductive dehalogenases, *pceA*, *tceA*, and *vcrA* (148, 175). Accompanied by advances in genome sequencing techniques, putative reductive dehalogenases in *Dehalococcoides* have been reported (148, 158, 198). Multiple reductive dehalogenase genes may be induced by a single chlorinated ethene in a microbial enrichment culture containing *Dehalococcoides*, as demonstrated by Futamata *et al.* (55). The X-ray crystal structure of PceA from *Sulfurospirillum multivorans* has been reported by Bommer *et al.* (20), and revealed that cobalamin supports reductive dechlorination.

In engineering practices associated with the bioremediation of chlorinated ethenes, electron donors (*e.g.* lactate, methanol, molasses, hydrogen release compounds, and vegetable oils) and vitamin B_12_ are commonly injected into contaminated sites in order to stimulate reductive dechlorination (144, 182). Yeast extract also stimulates reductive dechlorination (122). As for bioaugmentation, microbial consortia containing *Dehalococcoides*, such as KB-1 (45), have been introduced into contaminated sites. Successful case studies on bioaugmentation have been reported (48, 110). The density of useful microorganisms is used as a criterion for selecting biostimulation or bioaugmentation, and genetic biomarkers such as the *Dehalococcoides* 16S rRNA gene and reductive dehalogenase genes including *tceA*, *vcrA*, and *bvcA* are used as indicators (75, 182).

## Biodegradation of BTEX

### Aerobic biodegradation of BTEX

**Fig. 3:**
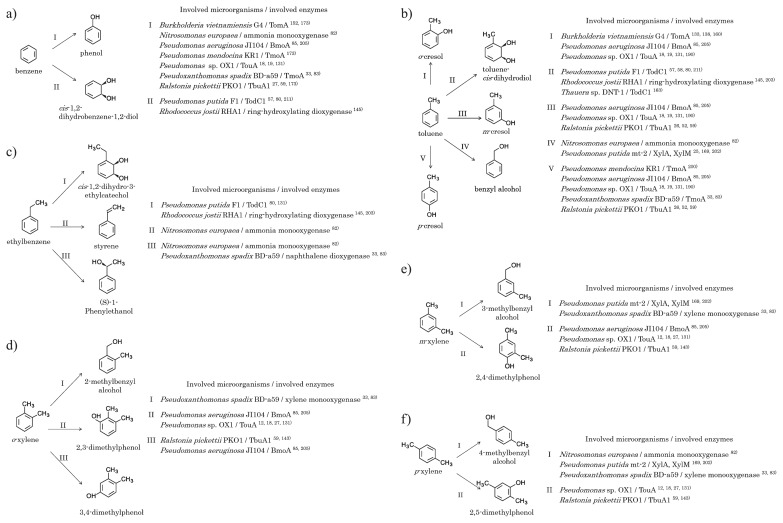
Possible initial steps in the aerobic biodegradation of benzene, toluene, ethylbenzene, and xylene. Each figure shows initial steps for a particular VOC: a), benzene; b), toluene; c), ethylbenzene; d), *o*-xylene; e), *m*-xylene; f), *p*-xylene. Abbreviations of involved enzymes indicate the following: TomA, toluene 2-monooxygenase; TmoA, toluene-4-monooxygenase; BmoA, benzene monooxygenase; TouA, toluene/*o*-xylene monooxygenase; TbuA1, toluene 3-monooxygenase; TodC1, toluene 2,3-dioxygenase; XylA, xylene monooxygenase; XylM, xylene monooxygenase.

The aerobic biodegradation of BTEX has a long history, and BTEX-degrading pathways may be traced back to the 1960s (57, 58). BTEX are oxidized by oxygenases (Fig. 3). The intermediates, catechol compounds, are produced by these pathways: catechol during benzene and toluene degradation, 3-methylcatechol during toluene, *o*-xylene, and *m*-xylene degradation, and 4-methylcatechol during *p*-xylene degradation.

The degradability of BTEX and the degrading pathway used by microorganisms depend on the types of degrading enzymes. *Pseudomonas mendocina* KR1, *Ralstonia pickettii* PKO1, and *B. vietnamiensis* G4 degrade benzene as well as toluene using toluene-4-monooxygenase (TmoA), toluene 3-monooxygenase (TbuA1), and toluene 2-monooxygenase (TomA), respectively (26, 52, 59, 138, 160, 161, 173, 200). *Pseudomonas* sp. OX1 degrades benzene, toluene, and *o*-xylene using the toluene/*o*-xylene monooxygenase TouA (12, 18, 19, 131, 190). *P. putida* mt-2 degrades toluene and xylenes using the xylene monooxygenases XylA and XylM (25, 159, 169, 202). *Pseudomonas aeruginosa* JI104 degrades benzene with the benzene monooxygenase BmoA (84, 85, 205). BmoA has low substrate specificity, and attacks toluene, xylene, and ethylbenzene, as well as benzene. *Pseudoxanthomonas spadix* BD-a59 degrades all BTEX (83), and has genes encoding TmoA, a xylene monooxygenase, and naphthalene monooxygenase (33). *Nitrosomonas europaea* degrades benzene, toluene, ethylbenzene, and *p*-xylene with an ammonia monooxygenase (82). In addition to monooxygenases, dioxygenases degrade BTEX. Toluene 2,3-dioxygenase (TodC1) from *P. putida* F1 degrades benzene, toluene, and ethylbenzene (57, 80, 131, 211, 212). *R. jostii* RHA1 degrades benzene, toluene, ethylbenzene, and *o*-xylene with a biphenyl dioxygenase and/or an ethylbenzene dioxygenase (145, 203). *Thauera* sp. DNT-1 degrades toluene with a dioxygenase under aerobic conditions (163). Strain DNT-1 also degrades toluene under anaerobic conditions via a pathway that produces benzyl succinate.

In the biostimulation of BTEX, an injection of oxygen release compounds (30) and an air sparging technique are commonly used in practical sites (79, 204). Various primer sets for PCR to detect genes coding BTEX-degrading enzymes have been developed (14, 68) and reverse-transcriptase (RT)-quantitative PCR for these genes is used in order to judge the effectiveness of oxygen injections (15).

### Anaerobic biodegradation of BTEX

The anaerobic biodegradation of BTEX was regarded as difficult for a long time, and the microbial transformation of xylenes under anoxic conditions was first confirmed in the mid-1980s (96). In addition to xylenes, the biodegradation of aromatic compounds such as benzene, toluene, and ethylbenzene, in the absence of oxygen has been reported since the 1990s (*e.g.* 44, 97, 151). During the anaerobic biodegradation of BTEX, aromatic compounds supply electrons to various electron acceptors such as NO_3_^−^, Fe^3+^, SO_4_^2−^, and HCO_3_^−^ (194, 199). The anaerobic degradation of toluene, as well as xylenes and ethylbenzene, starts with fumarate addition. In addition to fumarate addition, ethylbenzene is oxidized by a dehydrogenase that is produced by nitrate-reducing bacteria (16). Regarding the anaerobic degradation of benzene, the degradation pathway remains unclear; however, possible pathways have been proposed in previous reviews (37, 53, 194, 199).

Various anaerobic BTEX degraders have been isolated (*e.g.* 199). Among them, those using nitrate as an electron acceptor, such as *Aromatoleum aromaticum* EbN1 (151), *Azoarcus* sp. T (44), and *Thauera aromatica* K172 (5), have been isolated most frequently. In addition, microorganisms that use ferric iron and sulfate as electron accepters, such as *Geobacter grbiciae* TACP-2T (36) and *Desulfobacula toluolica* Tol2 (150), have also been isolated. Under methanogenic conditions, members of *Desulfobacterales* and *Coriobacteriaceae* are involved in the anaerobic degradation of benzene, which has been confirmed by stable isotope probing (140). Microorganisms that degrade *p*-xylene were only recently isolated; *Desulfosarcina* sp. PP31 was isolated as a degrader under sulfate-reducing conditions by Higashioka *et al.* (69). In the anaerobic toluene degradation pathway, the initial step, fumarate addition to toluene, is catalyzed by a benzyl succinate synthase (BssA) (100). BssA may also catalyze fumarate addition to *m*-xylene (1, 17).

## Biodegradation of chlorinated methanes

### Aerobic biodegradation of chlorinated methanes

Although the aerobic biodegradation of carbon tetrachloride (CT) remains uncertain, chloroform (CF) and dichloromethane (DCM) may be degraded under aerobic conditions. Methane, toluene, and butane monooxygenases oxidize CF to phosgene through trichloromethanol (28). Aerobic growth-linked DCM degradation mainly relies on glutathione, and DCM is dechlorinated and transformed to formaldehyde (127). The aerobic oxidation of DCM also occurs when methane and ammonia co-exist, although the degrading microorganisms do not assimilate DCM (142, 189).

Aerobic CF degraders have been obtained, as reported by Cappelletti *et al.* (28). Microorganisms, such as *M. trichosporium* OB3b (142), *Nocardioides* sp. CF8 (63), and *P. mendocina* KR1 (200), which degrade CF, use methane, butane, and toluene, respectively, as carbon and energy sources. An aerobic DCM-dechlorinating bacterium, *Methylopila helvetica* DM1, was first reported by Brunner *et al.* (23). A wide variety of methylotrophic bacteria, such as *Ancylobacter*, *Bacillus*, *Chryseobacterium*, *Hyphomicrobium*, and *Methylobacterium* (127) species, have been shown to degrade DCM with growth. *Rhodococcus* sp. EH831 degrades DCM and BTEX (99), suggesting that it has potential as a degrader of multiple VOCs. Most of these degrading microorganisms have been assessed for the presence of the DCM dehalogenase DcmA, which catalyzes the dechlorination of DCM. *Methylobacterium extorquens* DM4 is considered to have acquired the *dcmA* gene through horizontal gene transfer (156). In *M. extorquens* DM4, the acquired *dcmA* gene has been shown to participate in enzymatic or metabolic pathways, such as stress responses, metabolic tuning, regulation, cell structure adjustments to the solvent properties of DCM, DNA repair following damage with mutagenic agents, and chloride export (81, 120, 128). In addition, microbes that degrade DCM as non-growth substrates have also been isolated. *M. trichosporium* OB3b and *N. europaea* degrade DCM using a methane monooxygenase and ammonia monooxygenase, respectively (142, 189).

### Anaerobic biodegradation of chlorinated methanes

CT is dechlorinated under anaerobic conditions, and this process is mediated by cofactors such as corrinoid (93), coenzyme F430 (92), iron compounds (147), cytochromes (29), and humic substances (114). Under sulfate-reducing conditions, CT is mainly degraded to CS_2_ with the cofactor vitamin B_12_, a type of corrinoid, while it is degraded to CF in the absence of vitamin B_12_ (87). The dechlorination of CF to DCM occurs with or without growth. The growth-linked dechlorination of CF was first reported by Grostern *et al.* (61), and, in their study, *Dehalobacter* appeared to dechlorinate CF to DCM. The pathway of anaerobic DCM biodegradation remains unknown. Rather than being dechlorinated, DCM is considered to be fermented into formate and acetate (105).

Although anaerobic CT degraders have been isolated (146), the microorganisms that use CT as a carbon source have not. Acetogens, iron reducers, and methanogens degrade CT with cofactors. An acetogenic microorganism, *Acetobacterium woodii* DSM1030, anaerobically degrades CT and CF with vitamin B_12_ (46). Iron-reducing microorganisms, such as *Geobacter metallireducens* and *G. sulfurreducens*, degrade CT with iron compounds (109). Chloroform-reductive dehalogenases that are involved in CF degradation with growth have recently been revealed from *Dehalobacter* sp. CF50 (170, 172) and *Desulfitobacterium* sp. PR (42). As anaerobic DCM degraders, *Dehalobacterium formicoaceticum* DMC (104) and *Dehalobacter* strains (76) have been successfully isolated; however, the enzymes involved in the fermentative degradation of DCM have yet to be identified. In addition to degrading DCM under aerobic conditions, *Hyphomicrobium* sp. DM2 also degrades DCM using DcmA under anaerobic conditions (90).

## Interactions among co-existing VOCs

**Fig. 4:**
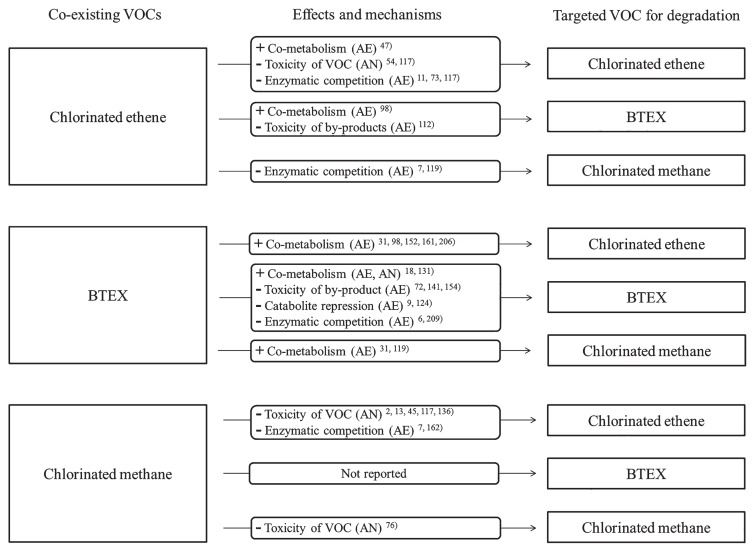
Possible interaction among the targeted VOC for degradation and co-existing VOCs. BTEX means benzene, toluene, ethylbenzene, and xylene. + and − indicate enhancement and constraint, respectively. AE and AN in brackets mean the effects occurring under aerobic and anaerobic conditions, respectively.

VOC biodegradation may be enhanced (207), constrained (141), and/or unaffected (24) by co-existing VOCs. In most cases, the enhancement of VOC degradation occurs because of the co-metabolism of VOC-degrading enzymes. Conversely, constraints of VOC degradation occur because of the toxicity of co-existing VOCs and their degradation products, catabolite repression, and competition with VOC-degrading enzymes (Fig. 4). We analyze the interactions among co-existing chlorinated ethenes, BTEX, and chlorinated methanes below.

## Enhancement

### Co-metabolism of multiple VOCs

Co-metabolism is defined as the transformation of an organic compound by a microorganism that is unable to use the compound as a source of energy or one of its constituent elements (3, 4). The co-existence of multiple VOCs may lead to co-metabolism in which one VOC is degraded as a growth-linked substrate and the other is co-metabolically degraded as a non-growth substrate. In co-metabolism, VOCs may be degraded by the same enzymes or one VOC functions as an inducer for the degradation of the other VOCs. Other VOC-degrading enzymes may be gratuitously induced by growth-linked substrates or their metabolites.

As described earlier, chlorinated ethenes are known to be degraded under aerobic conditions while degrading microorganisms utilize another chlorinated ethene, benzene, toluene, or xylene as the growth-linked substrate (Table 2). Degrading enzymes for BTEX, such as TouA, work on multiple BTEX in some cases, while BTEX are utilized as a growth-linked or non-growth substrate (18, 131).

The chlorinated methane, CF, is degraded as a non-growth substrate under aerobic conditions with a growth-linked substrate such as toluene and *o*-xylene (31, 119).

## Constraints

### Toxicity of co-existing VOCs

The toxicity of VOCs to microorganisms is caused by their inability to detoxify VOCs. The toxicity of VOCs influences microbial growth (88) and the degradability of VOCs (54). These effects are generally greater at high VOC concentrations (13, 45, 88). Tolerance to the toxicity of VOCs differs among microorganisms. Koenig *et al.* (88) reported that fast-growing microorganisms in VOC-free cultures, such as *Klebsiella* spp., have a higher tolerance to VOCs than *Desulfovibrio vulgaris*.

The constraints caused by the toxicity of co-existing VOCs occur in the anaerobic degradation of chlorinated ethenes (Table 3). In addition, the co-existence of chlorinated methanes inhibits the anaerobic degradation of chlorinated ethenes. During the anaerobic degradation of DCM, CF-mediated inhibition occurs, and this is attributed to its toxicity (76).

### Toxicity of by-products following the degradation of co-existing VOCs

When multiple VOCs co-exist, the toxicity of their by-products may affect the degradation of other VOCs. The by-products of VOC degradation, such as epoxide compounds and catechol compounds, are toxic. Epoxide compounds, which may be toxic to microorganisms and inhibit VOC degradation, are produced from the aerobic degradation of chlorinated ethenes (188). Of a mixture of four toluene-degrading bacteria, *P. putida* mt-2, *P. putida* F1, *P. putida* GJ31, and *B. vietnamiensis* G4, only *P. putida* mt-2 survived exposure to TCE and subsequent TCE degradation (112). This was because the other three microorganisms degraded TCE and then died because of the toxicity of the TCE by-product. In order to avoid the toxicity of epoxide compounds, a system, such as the epoxyalkane:coenzyme M transferase (EaCoMT) of *Mycobacterium* sp. JS60 (39, 40), is required to metabolize and/or detoxify by-products. The *etnE* gene, which encodes EaCoMT, is distributed in various environments, and has been detected in *Mycobacterium*, *Nocardioides*-like microorganisms, and *Haliea*-like microorganisms (101).

Catechol compounds are the main by-products of BTEX degradation, and concerns have been expressed regarding their toxicity (130). *P. putida* PPO1 produces toxic by-products, such as catechol compounds, during the degradation of *p*-xylene in the presence of benzene (141). The by-products from *p*-xylene inhibit benzene degradation, and the accumulation of these by-products increases the inhibition of VOC degradation. 3-Methylcatechol is produced in the degradation pathway of toluene, *o*-xylene, and *m*-xylene. Microbial growth ceases with the accumulation of 3-methylcatechol and toluene degradation is limited by *P. putida* strains (72, 154). In order to avoid constraints, microorganisms need enzymes, such as catechol 2,3-dioxygenase encoded by *xylE* of *P. putida* mt-2 (74, 202) and 3-methylcatechol 2,3-dioxygenase encoded by *todE* of *P. putida* F1 (21, 211), which degrade 3-methylcatechol.

### Catabolite repression

Catabolite repression occurs when microbes are exposed to multiple carbon sources. This leads the microorganisms to use a rapidly metabolizable carbon source first. Catabolite repression has been extensively studied in *Escherichia coli*, which uses glucose and other carbon sources (41), and, thus, catabolite repression may occur in the presence of multiple VOCs.

The degradation of toluene and xylene is inhibited by catabolite repression, which is induced by a rapidly metabolizable carbon source, such as succinate (8), a by-product of benzene and toluene degradation. The phosphotransferase enzyme IIA component encoded by the *pstN* gene, as well as the catabolite repression control (Crc) protein, is involved in this repression (9, 123). The Crc protein produced by *P. putida* has been studied in detail, and regulates toluene and xylene degradation by binding the translation initiation sites of mRNAs that are in the toluene/xylene degradation pathway (125). The mRNA levels of toluene/xylene degradation pathway genes, such as *xylA* and *xylM*, are more than 50% lower in a wild-type *P. putida* strain than in a *crc* mutant. Two small RNAs, corresponding to the *crcY* and *crcZ* genes, control Crc protein levels (126). Crc also inhibits the degradation of the by-product of toluene, benzoate, to catechol (124). These findings suggest that the presence of multiple VOCs leads to an excess of easily metabolizable carbon sources, as well as VOC by-products, which may cause catabolite repression and inhibit VOC degradation.

### Competition for degrading enzymes

Degrading enzymes work on different co-existing VOCs in some cases (Table 4). Methane monooxygenases degrade chlorinated ethenes and chlorinated methanes (43, 73, 142), and toluene monooxygenases also degrade chlorinated ethene and chlorinated methane compounds such as TCE and CF (119, 161). The oxygenases of BTEX react with multiple compounds of BTEX (57, 80). Thus, these enzymes compete for substrates.

## Future perspectives

Previous studies on VOC biodegradation mostly examined the degradation of a single VOC, even though contaminated sites are often polluted with multiple VOCs. In this review, a systematic survey associated with the biodegradation of chlorinated ethenes, BTEX, and chlorinated methanes was performed. The enhancement and constraint of VOC degradation were discussed with an emphasis on the effects of co-existing VOCs on useful microorganisms for a certain VOC. This review may provide fundamental, but useful knowledge for developing novel approaches to the biodegradation of multiple VOCs. There are diverse interactions among co-existing VOCs, depending on the kinds of degrading microorganisms and types of VOCs. In order to achieve effective designs and operations associated with the bioremediation of multiple VOCs in practice, the use of combined multiple microorganisms that degrade VOC and/or the introduction of microorganisms that degrade multiple VOCs may be a feasible strategy. Further studies on the interactions among VOCs are required, particularly on stimulatory interactions for increasing the efficiency of bioremediation. The use of new tools, such as isotopic and enzymatic analyses, will increase our understanding of the detailed mechanisms associated with interactions among co-existing VOCs.

## Figures and Tables

**Table 1:** Mechanisms associated with the initial step in the biodegradation of each type of VOC.

Category	Compounds	Aerobic degradation	Anaerobic degradation
Chlorinated ethenes	Tetrachloroethene (PCE)	Oxidation*1	Reductive dechlorination^116^,164)
	Trichloroethene (TCE)	Oxidation^115^,201)	
	Dichloroethene (DCE) *cis*-dichloroethene (*cis*-DCE) *trans*-dichloroethene (*trans*-DCE) 1,1-dichloroethene (1,1-DCE)		
	Vinyl chloride (VC)		
BTEX	Benzene	Oxidation^57^,^58^,186)	*2
	Toluene		Fumarate addition199)
	Ethylbenzene		Oxidation/fumarate addition^16^,199)
	Xylene *o*-xylene *m*-xylene *p*-xylene		Fumarate addition^96^,199)
Chlorinated methanes	Carbon tetrachloride (CT)	*3	Reductive dechlorination^61^,146)
	Chloroform (CF)	Oxidation28)	
	Dichloromethane (DCM)	Dechlorination (glutathione substitution)127)	Fermentation105)

*1 The aerobic degradation of PCE is limited, except as described by Ryoo *et al.* (155).

*2 The mechanisms underlying the anaerobic degradation of benzene are unclear, although hydroxylation to phenol, methylation to toluene, and carboxylation to benzoate were proposed by Weelink *et al.* (199).

*3 The aerobic degradation of CT remains ambiguous.

**Table 2:** Enhancement of VOC degradation by co-metabolism.

Microorganism	Targeted VOC for degradation	Growth-linked VOCs	Possible degrading enzyme	Reference
*Burkholderia vietnamiensis* G4	TCE	benzene, toluene	TomA	(152, 161, 173)
*Pseudomonas mendocina* KR1	CF	toluene	TmoA	(119)
*Pseudomonas putida* F1	*o*-xylene	ethylbenzene	*1	(131)
*Pseudomonas* sp. ENVBF1	CF	toluene	*1	(119)
*Pseudomonas* sp. ENVCP5	CF	toluene	*1	(119)
*Pseudomonas* sp. OX1	TCE*2	toluene, *o*-xylene	TouA	(31, 131)
	1,1-DCE*2			(31, 131)
	CF*2			(31, 131)
	ethylbenzene*2			(18, 131)
	*m*-xylene*2			(18, 131)
	*p*-xylene*2			(18, 131)
*Ralstonia pickettii* PKO1	TCE	toluene	TbuA1	(98, 206)
*Ralstonia* sp. TRW-1	*cis*-DCE	VC	*1	(47)
	*trans*-DCE		*1	(47)

*1 Unidentified enzymes degrading growth-linked VOCs and/or enzymes induced by growth-linked VOCs or their metabolites may be related to degradation.

*2 The degradation of VOCs was confirmed with*Escherichia coli* JM109 (pBZ1260) expressing *touA*.

Abbreviations of VOCs indicate the following: TCE, trichloroethene; DCE, dichloroethene; VC, vinyl chloride; CF, chloroform. Abbreviations of degrading enzymes denote the following: TomA, toluene 2-monooxygenase; TmoA, toluene-4-monooxygenase; TouA, toluene/*o*-xylene monooxygenase; TbuA1, toluene 3-monooxygenase.

**Table 3:** Constraints of VOC degradation caused by the toxicity of co-existing VOCs to microorganisms.

Microorganism	Targeted VOC for degradation	Co-existing toxic VOCs	Concentration of co-existing toxic VOCs	Reference
*Dehalobacter* sp.	DCM	CF	42 μM	(76)
*Desulfitobacterium hafniense* Y51	PCE	*cis*-DCE	5 mM*1	(54)
	TCE		5 mM*1	(54)
*Sulfurospirillum multivorans*	PCE	*cis*-DCE	14 mM*2	(136)
		CT	100 μM*2	(136)
		CF	25 μM*2	(136)
		DCM	50 μM*2	(136)
Microcosm	PCE	CT	10 15 μM	(2)
	VC		10 15 μM	(2)
Microcosm	PCE	CT	19 μM	(13)
		CF	4 μM	(13)
Microcosm	VC	CF	2.5 μM	(45)
Microcosm	TCE	CF	1.6 μM	(117)

*1 Desulfitobacterium hafniense Y51 lost the pceA gene.

*2 The concentration indicates the inhibition of PCE dehalogenase activity by 50%.

Abbreviations of VOCs indicate the following: PCE, tetrachloroethene; TCE, trichloroethene; DCE, dichloroethene; VC, vinyl chloride; CT, carbon tetrachloride; CF, chloroform; DCM, dichloromethane.

**Table 4:** Constraints of VOC degradation caused by competition for degrading enzymes.

Microorganism	Degrading enzyme	VOCs causing competitive inhibition	Reference
*Methylocystis* sp. SB2	pMMO	TCE, *cis*-DCE, and VC	(73)
*Methylosinus trichosporium* OB3b	sMMO	TCE and *trans*-DCE	(11)
		*cis*-DCE and *trans*-DCE	
*Pseudomonas mendocina* KR1	toluene monooxygenase	TCE and CF*1	(119)
*Pseudomonas putida* F1	toluene dioxygenase	benzene and toluene	(209)
		toluene and *p*-xylene	
*Pseudomonas* sp. CFS-215	toluene dioxygenase	benzene and toluene	(6)
*Pseudomonas* sp. ENVBF1	toluene monooxygenase	TCE and CF*1	(119)
*Pseudomonas* sp. OX1	TouA	TCE and CF*2	(162)
Methanotrophic microcosm	methane monooxygenase	TCE and CF	(7)

*1 The co-existence of TCE inhibited the degradation of CF, while TCE degradation was not affected. Abbreviations of VOCs indicate the following: TCE, trichloroethene; DCE, dichloroethene; VC, vinyl chloride; CF, chloroform. Abbreviations of degrading enzymes denote the following: pMMO, particulate methane monooxygenase; sMMO, soluble methane monooxygenase; TouA, toluene/*o*-xylene monooxygenase; MMO, methane monooxygenase.

*2 The TCE degradation rate decreased from 82% to 57% because of the co-existence of CF, while CF degradation did not change.
